# Effect of optical aberrations on intraocular pressure measurements using a microscale optical implant in *ex vivo* rabbit eyes

**DOI:** 10.1117/1.JBO.23.4.047002

**Published:** 2018-04-12

**Authors:** Samuel J. Han, Haeri Park, Jeong Oen Lee, Hyuck Choo

**Affiliations:** a California Institute of Technology, Department of Medical Engineering, Pasadena, California, United States; b California Institute of Technology, Department of Electrical Engineering, Pasadena, California, United States

**Keywords:** medical devices, optics, aberration, light, glaucoma

## Abstract

Elevated intraocular pressure (IOP) is the only modifiable major risk factor of glaucoma. Recently, accurate and continuous IOP monitoring has been demonstrated *in vivo* using an implantable sensor based on optical resonance with remote optical readout to improve patient outcomes. Here, we investigate the relationship between optical aberrations of *ex vivo* rabbit eyes and the performance of the IOP sensor using a custom-built setup integrated with a Shack–Hartmann sensor. The sensor readouts became less accurate as the aberrations increased in magnitude, but they remained within the clinically acceptable range. For root-mean-square wavefront errors of 0.10 to 0.94  μm, the accuracy and the signal-to-noise ratio were 0.58±0.32  mm Hg and 15.57±4.85  dB, respectively.

## 1 Introduction

Glaucoma is a leading cause of irreparable blindness,^1^^,^^2^ but the underlying mechanism of its pathophysiological development and progression remains unclear. Because the major identifiable and manageable risk factor of the disease is elevated intraocular pressure (IOP), all glaucoma treatments focus on monitoring and reducing elevated IOP.^3^^–^^6^ However, IOPs are monitored only a few times a year in clinics using tonometry despite its critical role in glaucoma management. Recently, researchers have developed radio-frequency (RF)-based contact-lens IOP sensors for 24-h monitoring and implantable RF-based sensors for direct IOP and other physiological pressure measurements.^7^^–^^33^ In addition, an increasing number of optics-based IOP and other biological pressure monitoring have been demonstrated based on flexible photonic crystal,^34^ interferometry,^35^^–^^42^ aberrometry,^43^ microfluidic or micromechanical implants,^44^^,^^45^ and laser-excited fluorescence.^46^

**Fig. 1:**
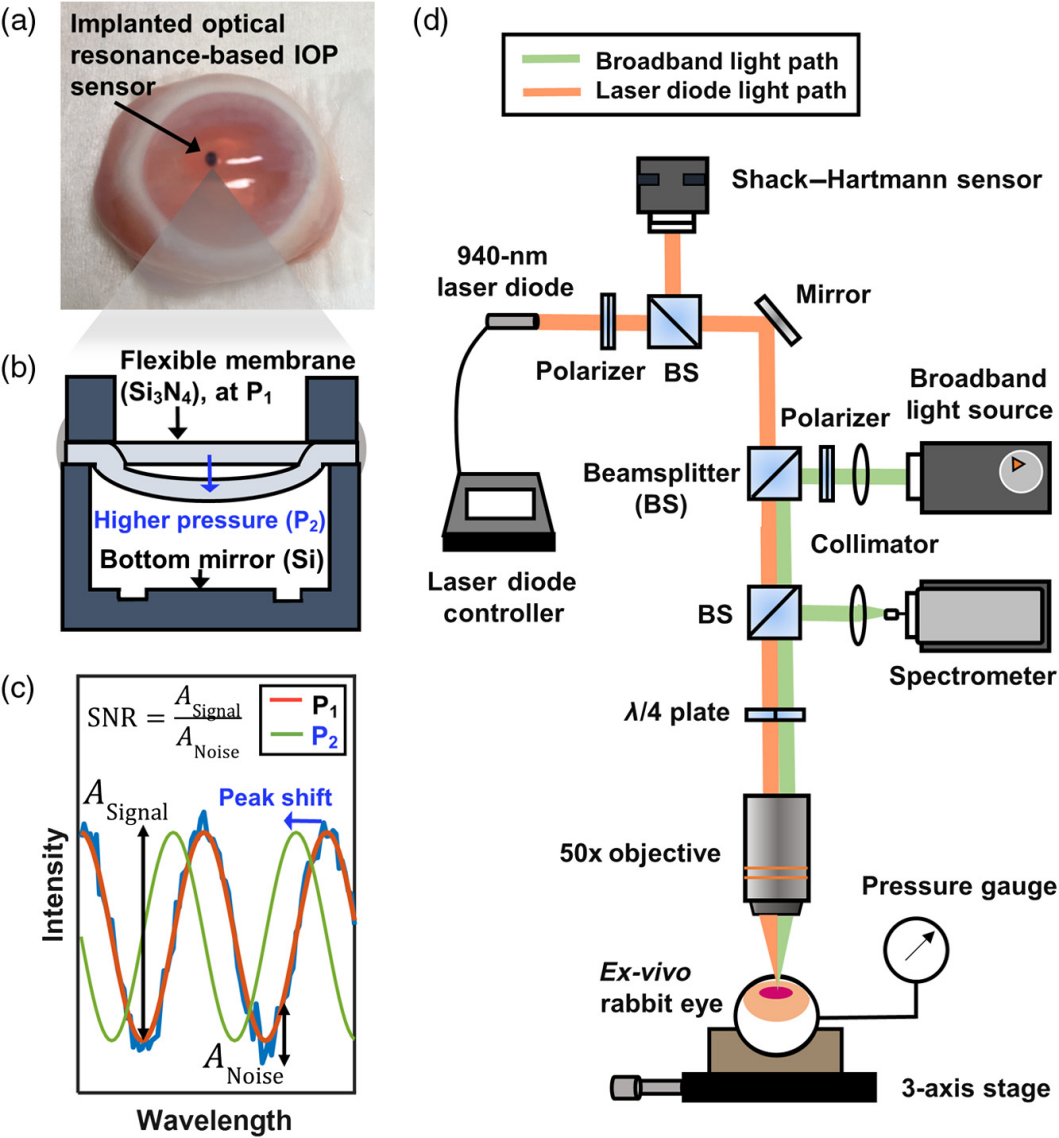
(a) A photograph of an *ex vivo* rabbit eye after IOP-sensor implantation. (b) A cross-sectional illustration of the hermetically sealed optical IOP sensor: an increase in IOP from $P_{1}$ to $P_{2}$ deflects the flexible membrane and changes the cavity’s optical resonance. (c) The optical resonance spectra reflected from the IOP sensor: the increase in IOP from $P_{1}$ to $P_{2}$ blueshifts the resonance, and there is one-to-one mapping between the resonance spectrum and the corresponding IOP. The accuracy is calculated by extracting the peak position, retrieving an IOP value, and comparing with the pressure-gauge readout. The SNR is calculated by extracting the amplitudes of the signal and noise and calculating the ratio between the two. (d) An illustration of the experimental setup for measuring optical aberrations and resonance spectra. Two light sources, the 940-nm laser diode (orange) and the broadband light source (green), share most of the optical path that includes an objective lens and an *ex vivo* rabbit eye.

To provide more accurate and frequent IOP monitoring and to improve treatment outcomes, researchers recently demonstrated implantable IOP sensors with remote optical readout in long-term *in vivo* studies.^47^^–^^49^ The sensor is a hermetically sealed micro-optical cavity with a top surface made of a flexible, transparent ${\mathrm{Si}}_{3}N_{4}$ membrane and a bottom surface made of a mirror-like silicon substrate. The top membrane deflects according to the ambient pressure, changing the resonance of the sensor cavity that is determined by the distance between the top membrane and the bottom mirror surface [Figs. 1(a)–1(c)]. When probed using broadband near-infrared (NIR) light, the sensor reflects an optical resonance spectrum made of peaks and valleys, the locations of which correlate with the present cavity gap and the corresponding IOP value [Fig. 1(c)]. Because NIR light is used to excite the cavity and obtain the pressure readout, the performance of the sensing system is inevitably influenced by optical aberrations that originate from the refractive-index profiles of the cornea and the anterior chamber of the eye. Although such optical aberrations and their influence on the performance of intraocular lenses have been characterized extensively using Shack–Hartmann (SH) sensors in previous clinical studies,^50^^–^^56^ the relationship between the performance of the implantable optical IOP sensor and ocular optical aberrations has yet to be studied. Clearly understanding the relationship between the ocular aberrations and the quality of IOP monitoring could ensure the effective and proper use of implantable optical IOP sensors across diverse patient groups.

In this study, we characterized the optical aberrations present in the corneas and anterior chambers of *ex vivo* rabbit eyes and examined their influence on the accuracy and signal-to-noise ratio (SNR) of IOP monitoring using optical-resonance-based sensor implants. The aberrated wavefronts of the optical reflection from an implanted sensor are related to the distortion and dislocation of a focal point, which in turn affects the accuracy and SNR of optical IOP monitoring. A customized setup was developed by coaxially integrating an SH sensor and a commercially available spectrometer to measure consecutively both the optical aberrations of the *ex vivo* eyes and the reflected optical spectra from the implanted IOP sensors [Fig. 1(d)]. First, we implanted a well-characterized reference chip coated with a thin photoresist film in *ex vivo* rabbit eyes and measured the optical spectra and the wavefront aberrations consecutively; doing so revealed the influence of the aberrations on the accuracy and SNR of the optical spectra obtained from the implanted reference chip. Next, we implanted the optical IOP sensors in *ex vivo* rabbit eyes, performed the same measurements, and determined the influence of the aberrations on the accuracy and SNR of IOP measurements. Finally, we investigated the effect of the position-dependent aberrations on IOP measurements by implanting the sensors at multiple locations inside an *ex vivo* rabbit eye and correlating IOP with spectral measurements.

## 2 Methods

### 2.1 Experimental Setup and Calibration

Figure 1(d) shows the experimental setup customized for *ex vivo* rabbit-eye measurements in this study. The setup enabled consecutive measurements of optical spectra and aberrations by incorporating two different systems: (1) a commercially available NIR spectrometer (Maya 2000; Ocean Optics, Dunedin, Florida) operating at 800 to 1100 nm with a broadband (400 to 1300 nm) halogen fiber-optic light source (OSL1; Thorlabs, Newton, New Jersey) for spectral resonance measurements and (2) a 150-μm-pitch CMOS-based SH sensor (WFS20-5C; Thorlabs, Newton, New Jersey) with a 940-nm laser diode (LP940-SF30; Thorlabs, Newton, New Jersey) for aberration characterization. Both the broadband light and the 940-nm laser were collimated and aligned coaxially to form focal points identical in size and location on the implant under measurement. The two distinct measurements, namely spectral and aberration, shared the same optical path, including an achromatic (690 to 1200 nm) quarter-wave plate (AQWP05M-980; Thorlabs, Newton, New Jersey), a 0.55-NA objective lens (50× M PLAN APO; Mitutoyo, Japan), the cornea of the *ex vivo* rabbit eye, the anterior chamber, and the active area of the sensor. A 0.55-NA objective provided a focal spot size that matched the active area of the sensor. Therefore, we were able to obtain the aberration data only from the active area of the sensor and the corneal area right above it.

Before use, the integrated SH sensor was precharacterized to remove any aberrations that could originate from the objective lens and other optical components in the experimental setup; this was done using a polished silicon chip immersed in water as a reflective surface for double-pass calibration. After calibration, the experimental setup showed an accuracy of 9.317 nm or approximately λ/101, which is very close to the accuracy of λ/100 specified by the manufacturer.

### 2.2 Measurements and Analysis

To study the influence of the optical aberrations on the sensor performance, we obtained one spectral and five aberration measurements in sequence and grouped them as one set. For the spectral measurement, we used only the broadband light source and collected an optical spectrum ranging from 800 to 1100 nm from an implant through the integrated spectrometer every 100 ms at a resolution of 0.22 nm. The sensor used in the experiments showed two peaks within the bandwidth of the collected spectrum. After collecting the optical spectra, we turned off the broadband light source and turned on the laser diode to perform five consecutive aberration measurements every 1 s at a frame rate of 79 fps. Between each set of measurements, we irrigated the *ex vivo* rabbit eyes with saline solution to maintain a uniform tear film.

We first focused on investigating the fundamental relationship between optical aberrations and the quality of the detected optical spectra using a reference chip that generated a well-characterized static optical spectrum similar to that of the implantable sensor. This eliminated the influence of ambient pressure or variation in the sensor geometry on the measurements. This reference chip was prepared by coating a silicon wafer with 15-μm-thick photoresist (Microchemicals GmbH, Germany) and dicing it into 1-mm×1-mm chips. The static, unchanging resonance came from the photoresist layer. Ten of these chips were inserted into 10 *ex vivo* rabbit eyes (Pel-Freeze, Rogers, Arkansas) through a 1-mm incision on the side of the corneas and placed at the center of the anterior chamber.

Next, we studied the effects of the aberrations on the quality of IOP readout using the aforementioned implantable IOP sensors with a micro-optical cavity. Before the experiments were performed, each sensor was characterized and calibrated using the 0.55-NA objective to remove any readout errors that could originate from the experimental setup. A sensor mounted on a flexible strip was implanted through a 3-mm incision on the side of the cornea and positioned at the center of the anterior chamber of 10 *ex vivo* rabbit eyes [Fig. 1(a)], whereupon we sutured the incision to prevent leakage. A commercially available high-accuracy (±0.26  mm Hg) digital pressure gauge (3584K11; McMaster-Carr, Elmhurst, Illinois) was connected to a 21-gauge needle that in turn was inserted directly into the anterior chambers of the *ex vivo* eyes to provide reference IOP values. The first five eyes (#1 to 5) were measured without injection of saline solution, whereas the last five eyes (#6 to 10) were injected with saline solution to emulate the IOP levels observed in living rabbits, resulting in IOP values of 16 to 20 mm Hg.

We also examined how the location of the IOP sensor in the eye would change the optical aberrations, which would in turn influence the accuracy and SNR of the IOP sensor. Three IOP sensors were implanted and positioned radially at three different locations inside the anterior chamber of an *ex vivo* rabbit eye. After the measurements of each eye, a reference chip or sensor was retrieved and rinsed in isopropyl alcohol and deionized water for reuse and the used *ex vivo* rabbit eye was disposed.

The optical spectra obtained from the sensor measurements were analyzed using a custom-built MATLAB™ signal processing algorithm that detects the location of the peaks and valleys and calculates the corresponding IOP value.^47^^,^^49^ The aberration-measurement data were processed using the software provided with the SH sensor (Thorlabs, Newton, New Jersey). We included tip and tilt in our analysis because they play an important role in the resonance of optical cavities. The root-mean-square (rms) wavefront error was calculated by averaging the values of the lowest 21 Zernike coefficients (representing up to the fifth Zernike order) obtained in the five consecutive measurements. The Zernike orders and terms used in our work are summarized in Table 1.^57^

**Table 1:** Zernike coefficients of the first five orders.^a^

Zernike order	Radial/azimuthal degree (n,m)	Expression	Aberration
First	(1,−1)	2ρ sin(θ)	Tilt
	(1, 1)	2ρ cos(θ)	Tip
Second	(2, 0)	$\sqrt{3}(2\rho^{2}-1)$	Defocus
	(2,−2)	$\sqrt{6}\rho^{2}\;\sin(2\theta )$	Oblique astigmatism
	(2, 2)	$\sqrt{6}\rho^{2}\;\cos(2\theta )$	Vertical astigmatism
Third	(3,−1)	$\sqrt{8}(3\rho^{3}-2\rho )\sin(\theta )$	Vertical coma
	(3, 1)	$\sqrt{8}(3\rho^{3}-2\rho )\cos(\theta )$	Horizontal coma
	(3,−3)	$\sqrt{8}\rho^{3}\;\sin(3\theta )$	Vertical trefoil
	(3, 3)	$\sqrt{8}\rho^{3}\;\cos(3\theta )$	Oblique trefoil
Fourth	(4, 0)	$\sqrt{5}(6\rho^{4}-6\rho^{2}+1)$	Primary spherical
	(4,−2)	$\sqrt{10}(4\rho^{4}-3\rho^{2})\sin(2\theta )$	Oblique secondary astigmatism
	(4, 2)	$\sqrt{10}(4\rho^{4}-3\rho^{2})\cos(2\theta )$	Vertical secondary astigmatism
	(4,−4)	$\sqrt{10}\rho^{4}\;\sin(4\theta )$	Oblique quadrafoil
	(4, 4)	$\sqrt{10}\rho^{4}\;\cos(4\theta )$	Vertical quadrafoil
Fifth	(5,−1)	$\sqrt{12}(10\rho^{5}-12\rho^{3}+3\rho )\sin(\theta )$	Vertical secondary coma
	(5, 1)	$\sqrt{12}(10\rho^{5}-12\rho^{3}+3\rho )\cos(\theta )$	Horizontal secondary coma
	(5,−3)	$\sqrt{12}(5\rho^{5}-4\rho^{3})\sin(3\theta )$	Vertical secondary trefoil
	(5, 3)	$\sqrt{12}(5\rho^{5}-4\rho^{3})\cos(3\theta )$	Oblique secondary trefoil
	(5,−5)	$\sqrt{12}\rho^{5}\;\sin(5\theta )$	Vertical pentafoil
	(5, 5)	$\sqrt{12}\rho^{5}\;\cos(5\theta )$	Oblique pentafoil

a From Ref. 57.

## 3 Results

### 3.1 Accuracy and SNR of Optical Spectra From the Reference Chip Implanted in Ex Vivo Eyes

**Fig. 2:**
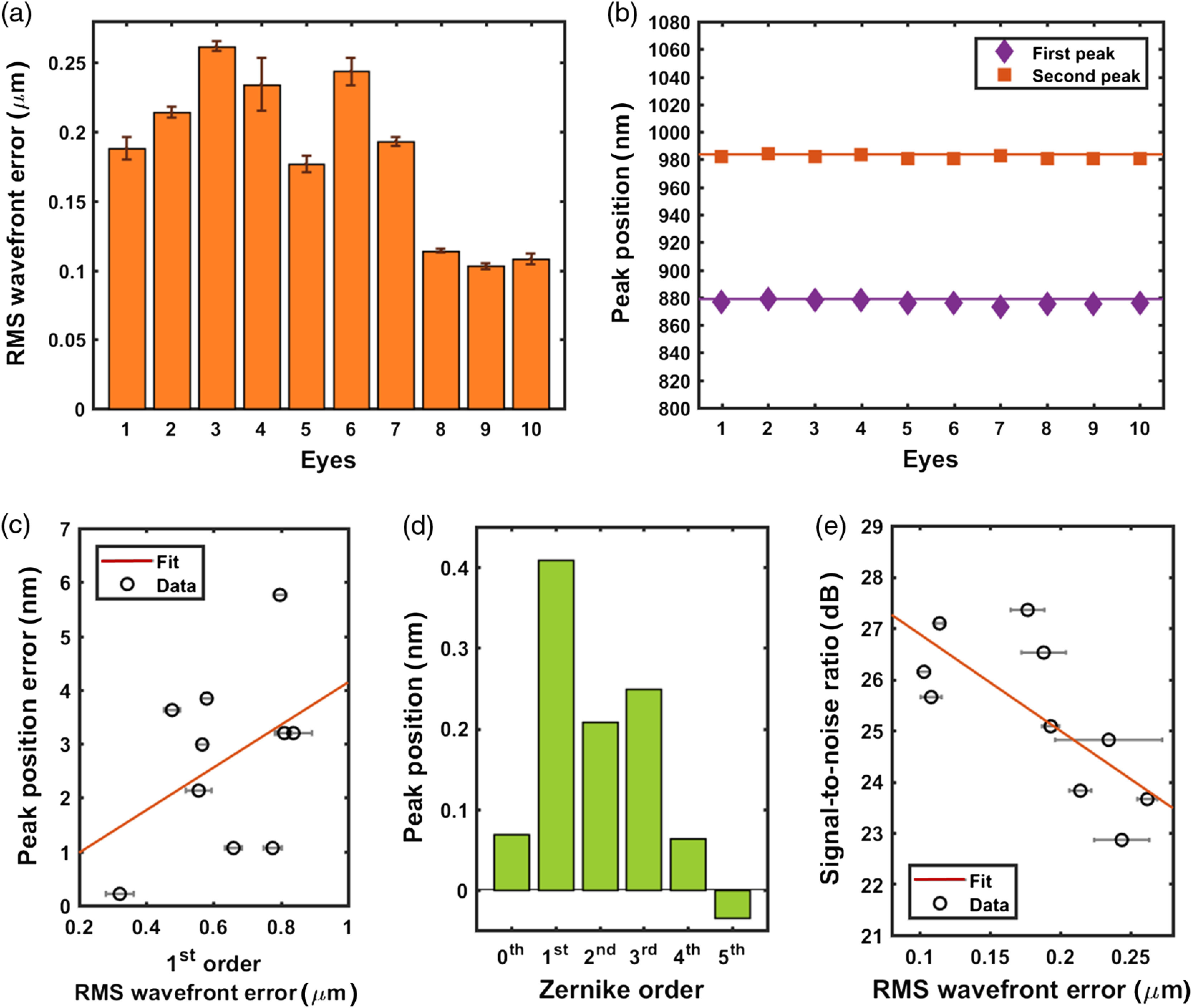
Measurements of reference chips implanted in 10 *ex vivo* rabbit eyes. (a) rms-wavefront error of the readouts from the reference chips. (b) Comparison between the peak locations in the optical spectra of a mock chip submerged in water (solid line) and implanted in *ex vivo* rabbit eyes (squares and diamonds). (c) The absolute errors in the measured peak locations plotted against the first-order Zernike rms-wavefront error. (d) Correlation coefficient of each Zernike order in relation to the error in the measured peak position. (e) SNR of the optical spectra of implanted reference chips plotted against the rms-wavefront error.

We evaluated the accuracy and SNR of the optical spectra reflected from the reference chips implanted in *ex vivo* rabbit eyes. Figure 2(a) shows the rms-wavefront error obtained in double-pass aberration measurements reflecting off the reference chips. The rms-wavefront errors were 0.10 to 0.26  μm. When spectral measurements were made on the reference chips implanted in *ex vivo* eyes, the locations of the resonance peaks deviated by 2.33±1.41  nm from the reference-chip measurements made in water [Fig. 2(b)]. We observed no significant correlation between the total rms-wavefront error and the deviation in resonance-peak positions. When we analyzed the deviation in terms of the rms-wavefront error of each Zernike order, the first-order Zernike coefficients (tip and tilt) had the strongest correlation to the peak-location error [Figs. 2(c) and 2(d)]. The SNR of the obtained spectra from the implanted reference chips showed a negative correlation with the rms-wavefront error, as shown in Fig. 2(e). We observed no single dominant Zernike coefficient that was strongly correlated with the SNR. Even the optical spectrum obtained in the presence of the highest rms-wavefront error (0.26  μm) still showed a robust SNR of 22.8 dB, well above the required 15-dB minimum SNR for IOP measurements.

### 3.2 Accuracy and SNR of Optical Spectra From an Optical Cavity Implant

**Fig. 3:**
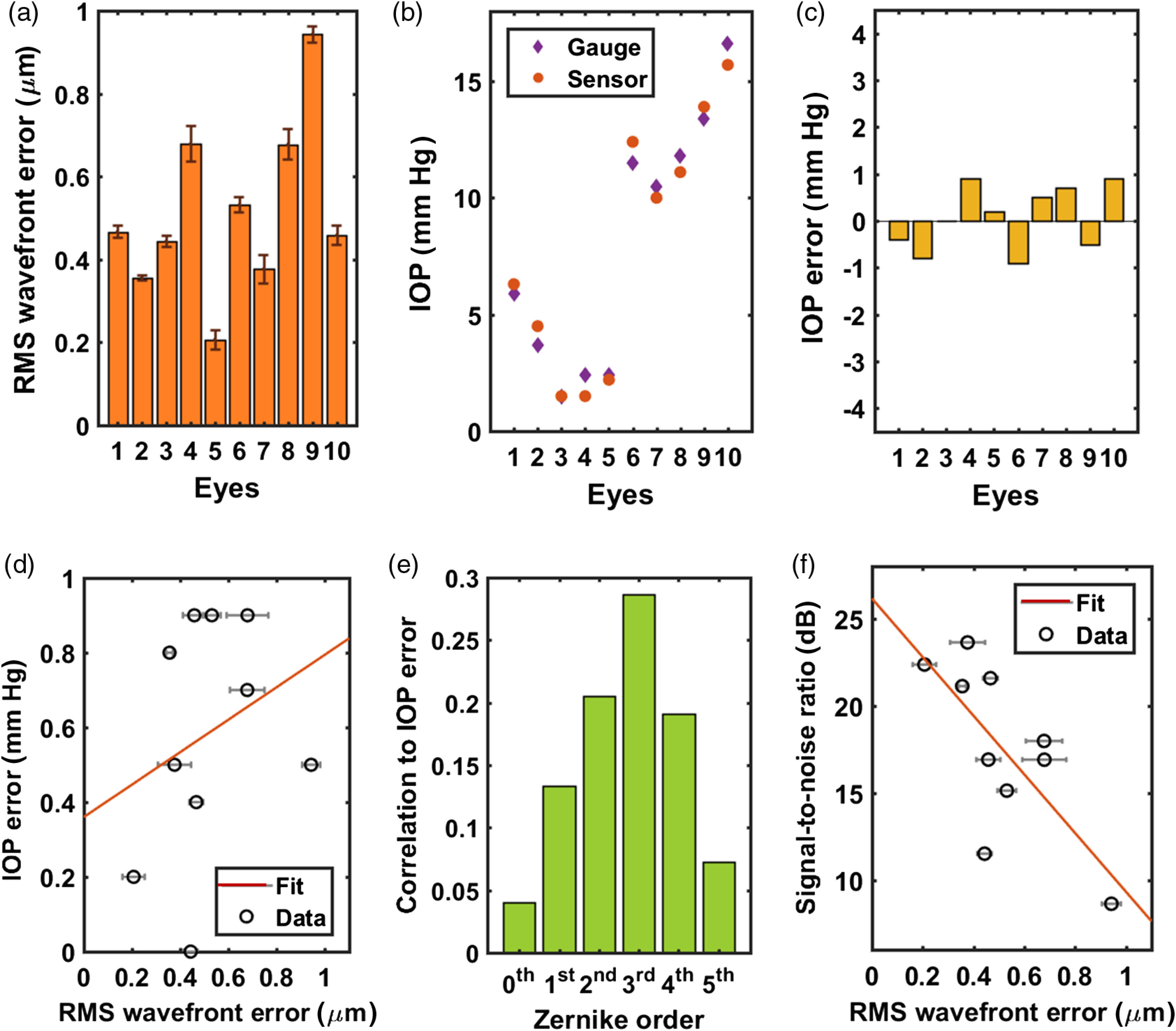
Aberrations, accuracy, and SNR measured using optical cavity implants in 10 *ex vivo* rabbit eyes. (a) rms-wavefront errors present in the reflection from implanted IOP sensors. Eyes #6 to 10 were injected with saline to increase the IOPs. (b) Comparison between the measured IOP values obtained from an implanted IOP sensor and a digital pressure gauge. (c) IOP error in the readouts from an IOP sensor. (d) Absolute IOP error plotted against the rms-wavefront error. (e) Correlation coefficient of each Zernike order in relation to the absolute IOP error. (f) SNR of the optical spectra of an implanted IOP sensor plotted against the rms-wavefront error.

We studied the accuracy and SNR of the optical resonant spectra from the optical-resonance-based IOP-sensing implants in 10 *ex vivo* rabbit eyes. While the pressure of five *ex vivo* rabbit eyes (#1 to 5) was maintained at their original values of <6  mm Hg, the other five *ex vivo* rabbit eyes (#6 to 10) were injected with saline, resulting in pressures of 10 to 17 mm Hg [Fig. 3(a)]. The rms-wavefront errors of the *ex vivo* eyes with saline injection were 0.38 to 0.94  μm, which were higher than the 0.21- to 0.68-μm range measured in the *ex vivo* eyes without injection [Fig. 3(b)]. The values of the IOP-readout error from the implanted sensors were smaller than ±1  mm Hg with an accuracy of 0.58±0.32  mm Hg [Fig. 3(c)], and the absolute values of the IOP error increased with rms-wavefront error, as shown in Fig. 3(d). The factor contributing most to the IOP error was the third-order Zernike term [Fig. 3(e)], which corresponds to comatic and trefoil aberrations. Figure 3(f) shows that the SNR of the optical resonance spectra reflected from the implanted sensors decreased with rms-wavefront error.

### 3.3 Accuracy and SNR of Optical Spectra From Optical Cavity Implants at Different Locations

**Fig. 4:**
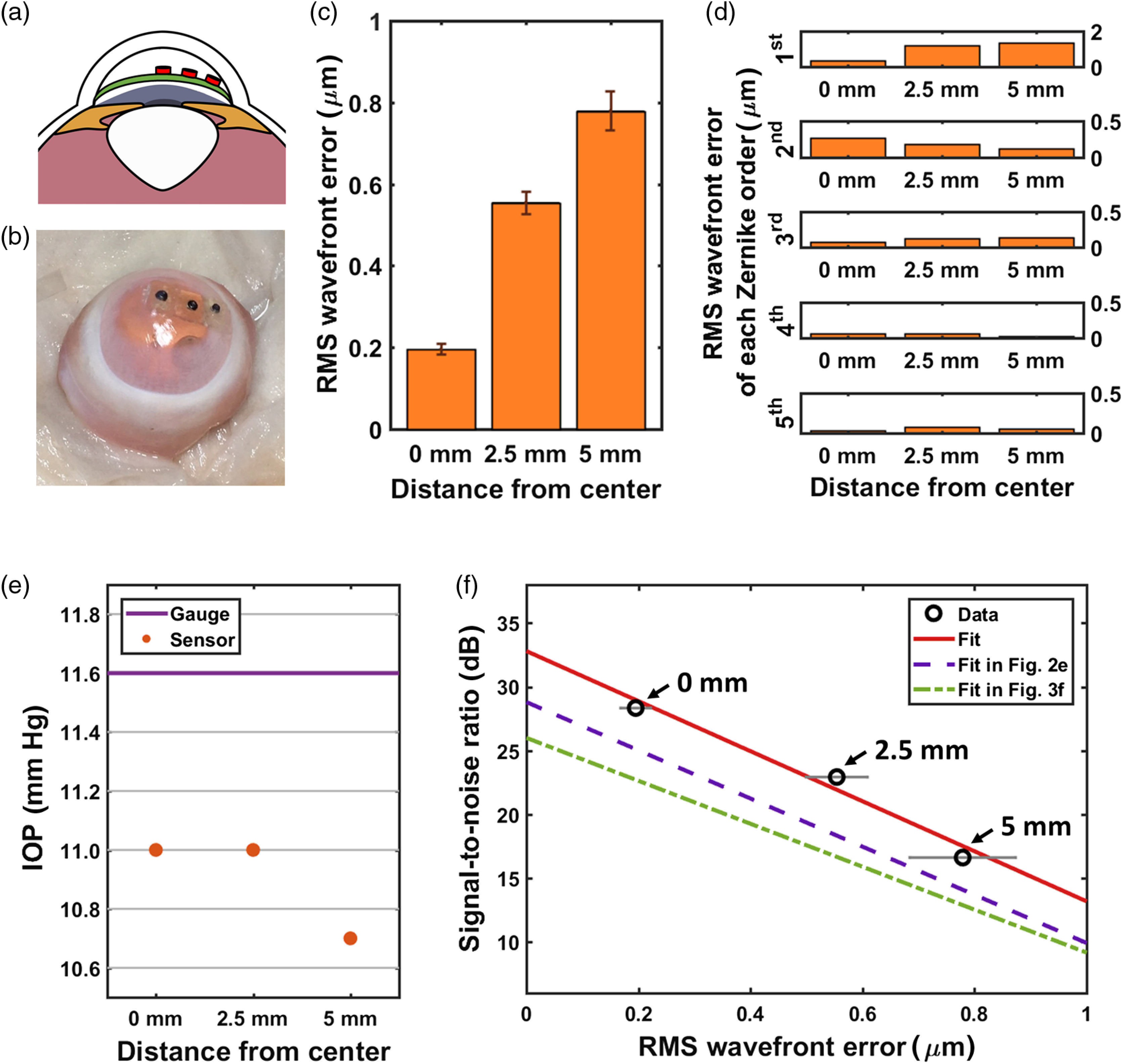
Measurements using three IOP sensors implanted at three different locations. (a) A cross-sectional illustration of the IOP sensors placed at three different locations. (b) A photograph of the three IOP sensors implanted inside the anterior chamber of an *ex vivo* rabbit eye. (c) The rms-wavefront error of the readout from each implanted IOP sensor. (d) rms-wavefront error of each Zernike order at different locations. (e) Comparison between the readings from the digital pressure gauge and the implanted IOP sensors. (f) SNR of the optical spectra of the implanted IOP sensors plotted against the rms-wavefront error.

We also investigated the accuracy of the IOP readouts and the SNR of the optical spectra when the sensors were placed at three different radial positions inside the anterior chamber of an *ex vivo* rabbit eye. A cross-sectional view of the three sensors placed at different positions is shown in Fig. 4(a). Three IOP sensors were attached to a strip at an interval of 2.5 mm, and the strip with the sensors was inserted through an incision and positioned inside the anterior chamber of an *ex vivo* rabbit eye [Fig. 4(b)]. The rms-wavefront error measured on each IOP implant is shown in Fig. 4(c). The rms-wavefront error increased with the distance from the implant to the center of the pupil or the optical axis of the eye. The first-order Zernike coefficients contributed the most to the larger rms-wavefront error observed at a greater distance from the center of the pupil [Fig. 4(d)]. The IOP readings from the three implanted sensors and the reference pressure gauge are plotted in Fig. 4(e). The sensor located at the farthest possible distance (5 mm) from the pupil center showed the largest IOP error of 0.9 mm Hg but still remained below 1 mm Hg, which is the clinically accepted maximum IOP error. In Fig. 4(f), the rms-wavefront error increased with the distance of the sensor from the center; consequently, the SNR of the optical spectra obtained from the IOP sensor decreased but stayed above 15 dB, which was the minimum SNR required for the sensor readout.

## 4 Discussion

### 4.1 Influence of Optical Aberrations on Accuracy of Optical IOP Readout

The magnitudes of the optical aberrations that we characterized in the *ex vivo* rabbit eyes were very close to the values reported previously in other studies.^58^ Our rms-wavefront errors from all measurements, which lie within 0.37±0.23  μm, are slightly larger than previously reported *in vivo* values, likely because of posthumous distortion of the eye geometry.

In the reference-chip testing, the first-order Zernike coefficients presenting tip and tilt showed the highest correlation (r=0.41) with erroneous shifts observed in the locations of the resonant peaks [Fig. 2(d)]. This was due to misalignment between the measurement setup and the reference chip. It was challenging to achieve perfect normal incidence between the optical axis of the measurement setup and the surface of the implanted reference chips because the precision of chip placement inside the *ex vivo* rabbit eyes was limited. The second- (defocus and astigmatism) and third-order (coma) Zernike coefficients were also correlated with the peak-location error, with the third-order coefficient being slightly more dominant. This is again attributed to the fact that defocus and coma are closely related to the precision of the optical alignment. It is also due to the dominance of coma in the native aberration of rabbit eyes.^58^ This outcome is consistent with the principles of thin-film interference or Fabry–Perot cavity resonance; the interference or resonance is heavily dependent on the optical path length, which is determined by the angle of incidence.^59^

A similar trend is observed between the rms-wavefront error of each Zernike order and the readout error of the IOP sensors [Fig. 3(e)], except that the first-order Zernike coefficients, namely tip and tilt, were not as dominant as in reference-chip testing. This indicates that we accomplished better measurement alignment when measuring the IOP sensors. Unlike the implanted reference chips with a $1\times 1\text{-}{\mathrm{mm}}^{2}$ flat reflective surface, the IOP sensors have a smaller, recessed sensing region made of two different surfaces that tend to be more angle sensitive [Fig. 1(b)]. This angle sensitivity requires better optical alignment during measurements. Therefore, the correlation between the first-order Zernike coefficients and the IOP error became relatively weak. The third-order rms-wavefront error, which represented coma, showed the strongest correlation with the IOP error, and its correlation magnitude of 0.29 was close to the 0.25 observed during the reference-chip measurements [Fig. 2(d)], indicating that the comatic aberrations that we observed in both cases were most likely a part of the native aberrations present in the *ex vivo* rabbit eyes.^58^ The second most contributing factor for the IOP error was the second-order rms-wavefront error, which represents defocus. In addition to the inherent second-order rms-wavefront error to the rabbit cornea, the deformable membrane of the sensor contributed to the increase in the second-order rms-wavefront error and its correlation to the IOP error. This trend is consistent with the previous study that showed that the second-order rms-wavefront error originating from a deflected membrane was more sensitive to IOP than the fourth- and higher-order rms-wavefront errors.^43^

When the sensors were implanted at three different radial distances from the pupil center in the anterior chamber [Figs. 4(a) and 4(b)], however, the first-order Zernike coefficients became the dominant factor that increased the total rms-wavefront error [Fig. 4(c)] and the IOP error [Fig. 4(e)]. As the IOP sensor became farther from the center of the pupil, the curvature of the cornea also increased and caused a larger degree of refraction at the interfaces, resulting in significant tip and tilt. Although the third-order Zernike coefficients show the same trend as the first-order ones, the magnitude of the first-order Zernike coefficients is much greater than that of the third-order ones. The dominance of the first-order Zernike coefficients explains the underestimated IOP readouts because of the redshift of the peaks from the sensors positioned farther away from the pupil center. Unlike the first- and third-order terms, the second- and fourth-order rms-wavefront errors decreased at a greater distance from the pupil center. This is partly attributed to the smaller defocus term in the second-order rms-wavefront error as the depth of the sensor location decreased and enabled better focusing. In addition, both the second- and fourth-order coefficients include astigmatism terms, which are negatively correlated with the depth of the sensor location. When a sensor is located closer to the cornea, the focused light illuminates through a smaller area on the cornea, thereby introducing less astigmatism.

### 4.2 Influence of Optical Aberrations on SNR of Optical Readout

The SNR of the optical spectra obtained from the reference-chip measurements was 22.8 to 27.5 dB [Fig. 2(e)], which exceeds the 7 to 23 dB of the IOP sensors as shown in Fig. 3(f). This is partly due to the larger measured/reflected area (1  mm×1  mm) of the implanted reference chips compared with that of an implanted IOP sensor (0.6 mm in diameter).

The major factor that decreased the SNR in both cases was the presence of the larger aberrations [Figs. 2(e) and 3(f)]. The larger aberrations came from the implant and/or the biomechanics of *ex vivo* rabbit eyes. The IOP sensor introduced extra aberration due to its flexible surface geometry.^43^ The native aberration of the sensors (0.168±0.014  μm) accounts for the increase in the average aberration in Fig. 3(b) (0.514  μm) when compared with that in Fig. 2(a) (0.184  μm). Additionally, the increase in IOP and the variation in the Young’s modulus of the corneas could result in the increase in the radius of corneal curvature and the increased aberration.^60^

The reduction in the SNR of the readouts under the greater rms-wavefront errors was observed in both reference-chip testing and IOP-sensor testing as well as in sensor-location testing that involved three sensors implanted at three different radial distances [Fig. 4(g)]. When compared with the SNR of the readouts in reference-chip testing and IOP-sensor testing [Figs. 2(e) and 3(f)], the SNR values obtained from this experiment [Fig. 4(f)] followed a similar fitting line to those used in Figs. 2(e) and 3(f). Furthermore, the dominance of the first Zernike order was observed in the case of sensor-location testing [Fig. 4(d)] but not in the case of IOP-sensor testing [Fig. 3(e)]. These outcomes indicate that the SNR of the readouts was primarily dependent on the total rms-wavefront error and was not particularly dependent on a single Zernike order. With no significant reduction in the SNR near the edge of the anterior chamber, this optical-resonance-based IOP-sensing implant is highly promising for use in human eyes without interrupting the vision.

## 5 Conclusion

We studied the effect of optical aberrations on the accuracy and the SNR of optical IOP monitoring obtained using ocular implants. Both reference chips and optical cavity implants showed high accuracy (<5.8  nm for a reference chip and <1  mm Hg with an optical cavity implant) along with good SNR (25.3±1.46  dB for a reference chip and 15.57±4.85  dB for an optical cavity implant) over rms-wavefront errors of 0.10 to 0.94  μm. Additionally, the IOP readout from the sensor that was located radially at 5 mm from the pupil center where one would expect the largest optical aberrations showed acceptable accuracy and SNR (<1  mm Hg and >15  dB). These results indicate that the use of optical cavity implants shows great promise for accurate and easy monitoring of IOP.

## References

[1] Quigley H. A. Broman A. T., “The number of people with glaucoma worldwide in 2010 and 2020,” Br. J. Ophthalmol. 90(3), 262–267 (2006). BJOPAL 0007-1161 10.1136/bjo.2005.081224 16488940 PMC1856963

[2] Tham Y. C. et al., “Global prevalence of glaucoma and projections of glaucoma burden through 2040: a systematic review and meta-analysis,” Ophthalmology 121(11), 2081–2090 (2014). 10.1016/j.ophtha.2014.05.013 24974815

[3] Quigley H. A., “Glaucoma,” Lancet 377(9774), 1367–1377 (2011). LANCAO 0140-6736 10.1016/S0140-6736(10)61423-7 21453963

[r4] Bengtsson B. et al., “Fluctuation of intraocular pressure and glaucoma progression in the early manifest glaucoma trial,” Ophthalmology 114(2), 205–209 (2007). 10.1016/j.ophtha.2006.07.060 17097736

[r5] De Moraes C. G. et al., “Risk factors for visual field progression in treated glaucoma,” Arch. Ophthalmol. 129(5), 562–568 (2011). AROPAW 0003-9950 10.1001/archophthalmol.2011.72 21555607

[6] Heijl A. et al., “Reduction of intraocular pressure and glaucoma progression: results from the early manifest glaucoma trial,” Arch. Ophthalmol. 120(10), 1268–1279 (2002). AROPAW 0003-9950 10.1001/archopht.120.10.1268 12365904

[7] Chen G. Z. Chan I. S. Lam D. C., “Capacitive contact lens sensor for continuous noninvasive intraocular pressure monitoring,” Sens. Actuators A 203, 112–118 (2013). 10.1016/j.sna.2013.08.029

[r8] Mansouri K. Shaarawy T., “Continuous intraocular pressure monitoring with a wireless ocular telemetry sensor: initial clinical experience in patients with open angle glaucoma,” Br. J. Ophthalmol. 95(5), 627–629 (2011). BJOPAL 0007-1161 10.1136/bjo.2010.192922 21216796

[r9] Faschinger C. Mossböck G., “Continuous 24 h monitoring of changes in intraocular pressure with the wireless contact lens sensor Triggerfish™. First results in patients,” Der Ophthalmologe: Zeitschrift der Deutschen Ophthalmologischen Gesellschaft 107(10), 918–922 (2010). 10.1007/s00347-010-2198-4 20535482

[r10] Leonardi M. et al., “A soft contact lens with a MEMS strain gage embedded for intraocular pressure monitoring,” in 12th Int. Conf. on TRANSDUCERS, Solid-State Sensors, Actuators and Microsystems, Vol. 2, pp. 1043–1046 (2003). 10.1109/SENSOR.2003.1216947

[r11] Mansouri K. et al., “Continuous 24-hour monitoring of intraocular pressure patterns with a contact lens sensor: safety, tolerability, and reproducibility in patients with glaucoma,” Arch. Ophthalmol. 130(12), 1534–1539 (2012). AROPAW 0003-9950 10.1001/archophthalmol.2012.2280 23229696

[r12] Greene M. Gilman B., “Intraocular pressure measurement with instrumented contact lenses,” Invest. Ophthalmol. Visual Sci. 13(4), 299–302 (1974). IOVSDA 0146-0404 4818815

[r13] Leonardi M. et al., “Wireless contact lens sensor for intraocular pressure monitoring: assessment on enucleated pig eyes,” Acta Ophthalmol. 87(4), 433–437 (2009). 10.1111/aos.2009.87.issue-4 19016660

[r14] Leonardi M. et al., “First steps toward noninvasive intraocular pressure monitoring with a sensing contact lens,” Invest. Ophthalmol. Visual Sci. 45(9), 3113–3117 (2004). IOVSDA 0146-0404 10.1167/iovs.04-0015 15326128

[r15] Huang Y. C. et al., “A contact lens sensor system with a microcapacitor for wireless intraocular pressure monitoring,” in IEEE Sensors, 2013, pp. 1–4 (2013). SNSRES 0746-9462 10.1109/ICSENS.2013.6688174

[r16] Farandos N. M. et al., “Contact lens sensors in ocular diagnostics,” Adv. Healthcare Mater. 4(6), 792–810 (2015). 10.1002/adhm.201400504 25400274

[r17] Ladage P. M. et al., “Pseudomonas aeruginosa corneal binding after 24-hour orthokeratology lens wear,” Eye Contact Lens 30(3), 173–178 (2004). 10.1097/01.ICL.0000133220.32701.C8 15499241

[r18] Imayasu M. et al., “The relation between contact lens oxygen transmissibility and binding of Pseudomonas aeruginosa to the cornea after overnight wear,” Ophthalmology 101(2), 371–388 (1994). 10.1016/S0161-6420(94)31326-1 8115159

[r19] Ren D. H. et al., “The relationship between contact lens oxygen permeability and binding of Pseudomonas aeruginosa to human corneal epithelial cells after overnight and extended wear,” CLAO J. 25(2), 81 (1999). CLAJEU 0733-8902 10344294

[r20] Kim J. et al., “Wearable smart sensor systems integrated on soft contact lenses for wireless ocular diagnostics,” Nat. Commun. 8, 14997 (2017). NCAOBW 2041-1723 10.1038/ncomms14997 28447604 PMC5414034

[r21] Koutsonas A. et al., “Implantation of a novel telemetric intraocular pressure sensor in patients with glaucoma (ARGOS study): 1-year results telemetric intraocular pressure sensor,” Invest. Ophthalmol. Visual Sci. 56(2), 1063–1069 (2015). IOVSDA 0146-0404 10.1167/iovs.14-14925 25613949

[r22] Todani A. et al., “Intraocular pressure measurement by radio wave telemetry,” Invest. Ophthalmol. Visual Sci. 52(13), 9573–9580 (2011). IOVSDA 0146-0404 10.1167/iovs.11-7878 22039243

[r23] Katuri K. C. Asrani S. Ramasubramanian M. K., “Intraocular pressure monitoring sensors,” IEEE Sens. J. 8(1), 12–19 (2008). ISJEAZ 1530-437X 10.1109/JSEN.2007.912539

[r24] Collins C. C., “Miniature passive pressure transensor for implanting in the eye,” IEEE Trans. Biomed. Eng. BME-14, 74–83 (1967). IEBEAX 0018-9294 10.1109/TBME.1967.4502474 6078978

[r25] Jang C. I. et al., “Effects of inner materials on the sensitivity and phase depth of wireless inductive pressure sensors for monitoring intraocular pressure,” Appl. Phys. Lett. 108(10), 103701 (2016). APPLAB 0003-6951 10.1063/1.4943136

[r26] Varel Ç. et al., “A wireless intraocular pressure monitoring device with a solder-filled microchannel antenna,” J. Micromech. Microeng. 24(4), 045012 (2014). JMMIEZ 0960-1317 10.1088/0960-1317/24/4/045012

[r27] Chow E. Y. Chlebowski A. L. Irazoqui P. P., “A miniature-implantable RF-wireless active glaucoma intraocular pressure monitor,” IEEE Trans. Biomed. Circuits Syst. 4(6), 340–349 (2010). 10.1109/TBCAS.2010.2081364 23850751

[r28] Xue N. Chang S. P. Lee J. B., “A SU-8-based microfabricated implantable inductively coupled passive RF wireless intraocular pressure sensor,” J. Microelectromech. Syst. 21(6), 1338–1346 (2012). JMIYET 1057-7157 10.1109/JMEMS.2012.2206072

[r29] Stangel K. et al., “A programmable intraocular CMOS pressure sensor system implant,” IEEE J. Solid-State Circuits 36(7), 1094–1100 (2001). IJSCBC 0018-9200 10.1109/4.933466

[r30] Rosengren L. et al., “A system for passive implantable pressure sensors,” Sens. Actuators A. 43(1–3), 55–58 (1994). SAAPEB 0924-4247 10.1016/0924-4247(93)00664-P

[r31] Ouda M. H. et al., “5.2-GHz RF power harvester in 0.18-μm CMOS for implantable intraocular pressure monitoring,” IEEE Trans. Microwave Theory Tech. 61(5), 2177–2184 (2013). 10.1109/TMTT.2013.2255621

[r32] Donida A. et al., “A circadian and cardiac intraocular pressure sensor for smart implantable lens,” IEEE Trans. Biomed. Circuits Syst. 9(6), 777–789 (2015). 10.1109/TBCAS.2015.2501320 26800549

[33] Chen P. J. et al., “Microfabricated implantable parylene-based wireless passive intraocular pressure sensors,” J. Microelectromech. Syst. 17(6), 1342–1351 (2008). JMIYET 1057-7157 10.1109/JMEMS.2008.2004945

[34] Karrock T. Gerken M., “Pressure sensor based on flexible photonic crystal membrane,” Biomed. Opt. Express 6(12), 4901–4911 (2015). BOEICL 2156-7085 10.1364/BOE.6.004901 26713204 PMC4679264

[35] Melamud R. et al., “Development of an SU-8 Fabry–Perot blood pressure sensor,” in 18th IEEE Int. Conf. on Micro Electro Mechanical Systems (MEMS ’05), pp. 810–813 (2005). 10.1109/MEMSYS.2005.1454053

[r36] Cibula E. Donlagic D. Stropnik C., “Miniature fiber optic pressure sensor for medical applications,” in Proc. IEEE Sensors, pp. 711–714 (2002). 10.1109/ICSENS.2002.1037190

[r37] Bae H. Yu M., “Miniature Fabry–Perot pressure sensor created by using UV-molding process with an optical fiber based mold,” Opt. Express 20(13), 14573–14583 (2012). OPEXFF 1094-4087 10.1364/OE.20.014573 22714519

[r38] Hill G. et al. “SU-8 MEMS Fabry–Perot pressure sensor,” Sens. Actuators A 138(1), 52–62 (2007). 10.1016/j.sna.2007.04.047

[r39] Wolthuis R. A. et al., “Development of medical pressure and temperature sensors employing optical spectrum modulation,” IEEE Trans. Biomed. Eng. 38(10), 974–981 (1991). IEBEAX 0018-9294 10.1109/10.88443 1761298

[r40] Li M. Wang M. Li H., “Optical MEMS pressure sensor based on Fabry–Perot interferometry,” Opt. Express 14(4), 1497–1504 (2006). OPEXFF 1094-4087 10.1364/OE.14.001497 19503474

[r41] Totsu K. Haga Y. Esashi M., “Ultra-miniature fiber-optic pressure sensor using white light interferometry,” J. Micromech. Microeng. 15(1), 71–75 (2004). JMMIEZ 0960-1317 10.1088/0960-1317/15/1/011

[42] Phan A. et al., “Design of an optical pressure measurement system for intraocular pressure monitoring,” IEEE Sens. J. 18(1), 61–68 (2018). ISJEAZ 1530-437X 10.1109/JSEN.2017.2767539

[43] Nazarov A. et al., “Assessment of intraocular pressure sensing using an implanted reflective flexible membrane,” J. Biomed. Opt. 22(4), 047001 (2017). JBOPFO 1083-3668 10.1117/1.JBO.22.4.047001 28384704

[44] Araci I. E. et al., “An implantable microfluidic device for selfmonitoring of intraocular pressure,” Nat. Med. 20(9), 1074–1078 (2014).1078-8956 10.1038/nm.3621 25150497

[45] Chen P. J. et al., “Impanatable micromechanical parylene-based pressure sensors for unpowered intraoular pressure sensing,” J. Micromech. Microeng. 17(10), 1931–1938 (2007). JMMIEZ 0960-1317 10.1088/0960-1317/17/10/002

[46] Ghannad-Rezaie M. et al., “A powerless optical microsensor for monitoring intraocular pressure with keratoprostheses,” in Transducers & Eurosensors XXVII: The 17th Int. Conf. on Solid-State Sensors, Actuators and Microsystems (TRANSDUCERS & EUROSENSORS XXVII ’13), Vol. 17, pp. 2708–2711 (2013). 10.1109/Transducers.2013.6627365

[47] Lee J. O. et al., “A microscale optical implant for continuous in-vivo monitoring of intraocular pressure,” Microsys. Nanoeng. 3, 17057 (2017). 10.1038/micronano.2017.57 PMC6445001 31057882

[r48] Lee J. O. et al., “Biocompatible multifunctional black‐silicon for implantable intraocular sensor,” Adv. Healthcare Mater. 6(4), 1601356 (2017). 10.1002/adhm.201601356 PMC5522682 28081305

[49] Kim K. H. et al., “Real-time in vivo intraocular pressure monitoring using an optomechanical implant and an artificial neural network,” IEEE Sens. J. 17(22), 7394–7404 (2017). ISJEAZ 1530-437X 10.1109/JSEN.2017.2760140 29422780 PMC5798645

[50] Platt B. C. Shack R., “History and principles of Shack–Hartmann wavefront sensing,” J. Refract. Surg. 17(5), S573–S577 (2001). JRSUEY 0883-0444 10.3928/1081-597X-20010901-13 11583233

[r51] Prieto P. M. et al., “Analysis of the performance of the Hartmann–Shack sensor in the human eye,” J. Opt. Soc. Am. A 17(8), 1388–1398 (2000). 10.1364/JOSAA.17.001388 10935866

[r52] Liang J. et al., “Objective measurement of wave aberrations of the human eye with the use of a Hartmann–Shack wave-front sensor,” J. Opt. Soc. Am. A 11(7), 1949–1957 (1994). 10.1364/JOSAA.11.001949 8071736

[r53] Thibos L. N. Xin H., “Clinical applications of the Shack–Hartmann aberrometer,” Optom. Vision Sci. 76(12), 817–825 (1999). OVSCET 1040-5488 10.1097/00006324-199912000-00016 10612402

[r54] Guirao A. et al., “Corneal optical aberrations and retinal image quality in patients in whom monofocal intraocular lenses were implanted,” Arch. Ophthalmol. 120(9), 1143–1151 (2002). AROPAW 0003-9950 10.1001/archopht.120.9.1143 12215087

[r55] Marcos S. Sergio B. Ignacio J. A., “Optical quality and depth-of-field of eyes implanted with spherical and aspheric intraocular lenses,” J. Refract. Surg. 21(3), 223–235 (2005). JRSUEY 0883-0444 10.3928/1081-597X-20050501-05 15977879

[56] Taketani F. et al., “Influence of intraocular lens tilt and decentration on wavefront aberrations,” J. Cataract Refractive Surg. 30(10), 2158–2162 (2004). 10.1016/j.jcrs.2004.02.072 15474830

[57] Thibos L. N. et al., “Standards for reporting the optical aberrations of eyes,” J. Refractive Surg. 18(5), S652–S660 (2002). JRSUEY 0883-0444 10.3928/1081-597X-20020901-30 12361175

[58] Chen L. et al., “Comparison of wavefront aberrations in rabbit and human eyes,” Clin. Exp. Optom. 97(6), 534–539 (2014). 10.1111/cxo.2014.97.issue-6 25069625

[59] Teich M. C. Salech B., Fundamentals of Photonics, 22nd ed., Wiley Interscience, New York (1991).

[60] Liu J. Roberts C. J., “Influence of corneal biomechanical properties on intraocular pressure measurement: quantitative analysis,” J. Cataract Refractive Surg. 31(1), 146–155 (2005). 10.1016/j.jcrs.2004.09.031 15721707

